# Diaphragm Postural Function Analysis Using Magnetic Resonance Imaging

**DOI:** 10.1371/journal.pone.0056724

**Published:** 2013-03-14

**Authors:** Pavel Vostatek, Daniel Novák, Tomas Rychnovský, Šarka Rychnovská

**Affiliations:** 1 Department of Cybernetics, Czech Technical University in Prague, Prague, Czech Republic; 2 AVETE OMNE Physiotherapy Center, Filmarska 19, Prague, Czech Republic; The University of Tennessee Health Science Center, United States of America

## Abstract

We present a postural analysis of diaphragm function using magnetic resonance imaging (MRI). The main aim of the study was to identify changes in diaphragm motion and shape when postural demands on the body were increased (loading applied to a distal part of the extended lower extremities against the flexion of the hips was used). Sixteen healthy subjects were compared with 17 subjects suffering from chronic low back pain and in whom structural spine disorders had been identified. Two sets of features were calculated from MRI recordings: dynamic parameters reflecting diaphragm action, and static parameters reflecting diaphragm anatomic characteristics. A statistical analysis showed that the diaphragm respiratory and postural changes were significantly slower, bigger in size and better balanced in the control group. When a load was applied to the lower limbs, the pathological subjects were mostly not able to maintain the respiratory diaphragm function, which was lowered significantly. Subjects from the control group showed more stable parameters of both respiratory and postural function. Our findings consistently affirmed worse muscle cooperation in the low back pain population subgroup. A clear relation with spinal findings and with low back pain remains undecided, but various findings in the literature were confirmed. The most important finding is the need to further address various mechanisms used by patients to compensate deep muscle insufficiency.

## Introduction

The diaphragm and deep stabilization muscles of the body have been described as an important functional unit for dynamic spinal stabilization [1], [2]. The diaphragm precedes any movement of the body by lowering and subsequently establishing abdominal pressure which helps to stabilize the lumbar part of the spine. Proper activation of the diaphragm within the stabilization mechanism requires the lower ribs to be in an expiratory (low) position. During the breathing cycle, the lower ribs have to stay in the expiratory position and only expand to the sides. This is an important assumption for the straight and stabilized spine. Under these conditions, the motion of the diaphragm during respiration is smooth, and properly helps to maintain abdominal pressure.

Dysfunction of the cooperation among diaphragm, abdominal muscles, pelvic floor muscles and the deep back muscles is the main cause of vertebrogenic diseases and structural spine findings such as hernia, spondylosis and spondylarthrosis [3], [4]. Diaphragm function control is a broad and important issue for a number of fields of investigation, including pulmonology [5], chest surgery [6], rehabilitation [7] and gastroenterology [8]. However, studies dealing with the lumbar stabilization system mostly do not include diaphragm activity monitoring [9]. A traditional objective of studies dealing with diaphragm function is the diaphragm respiratory function [10]; studies focused on postural function are rare.

Studies focused on diaphragm activation with the aim of posture stabilization include Hodges [11]–[14], who concluded phase modulation corresponding to the movement of the upper limbs in diaphragm electromyography records. Some works deal with various modes of diaphragm functions in various respiration types [15], [16] or in situations not directly related to respiration, e.g. activation during breath holding [17]. These studies have always concentrated on healthy subjects who did not exhibit symptoms of respiratory disease or vertebrogenic problems.

### The use of magnetic resonance imaging for diaphragm assessment

Studies dealing with diaphragm motion using MRI are taken as a valid method for intrathoracic movement investigations [18]. Gierada [19] assessed MRI artifacts and concluded that MRI is a valid method for diaphragm image processing. Gierada [20] also used MRI for observing the anteroposterior size of the thorax, the height of the diaphragm during inspiration and expiration, and also the ventral and dorsal costophrenic angle during maximal breathe in and out. Kotani [21] and Chu [22] assessed chest and diaphragm movements for scoliosis patients. Suga [23] compared healthy subjects and subjects with chronic obstructive pulmonary disease (COPD), measuring the height, excursions and antero-posterior (AP) size of the diaphragm with the zone of apposition. Paradox diaphragm movements for subjects with COPD were investigated by Iwasawa [10]. Iwasawa used deep breath sequences while comparing diaphragm height and costophrenic angles. The study consisted of healthy subjects and subjects with scoliosis. Kotani [21] concluded that there was ordinary diaphragm motion with limited rib cage motion in the scoliosis group. The position of the diaphragm was measured relative to the apex of the lungs to the highest point of the diaphragm. Chu [22] compared healthy subjects against subjects with scoliosis, finding the same amount of diaphragm movement for both groups. The scoliosis group had the diaphragm significantly lower in the trunk and relatively smaller lung volumes. The distance between the apex of the lungs and the diaphragm ligaments was measured by Kondo [24], comparing young and old subjects. The effect of intraabdominal pressure on the lumbar part of the spine was observed by MRI and pressure measurement by Daggfeldt and Thorstensson [25]. Differences in diaphragm movement while performing thoracic or pulmonary breathing with the same spirometric parameters were tested by Plathow [26]. Plathow also examined the vital capacity of the lungs compared with 2D and 3D views in [27]. He concluded that there was a better correlation between the lung capacity and the 3D scans. In another study, Plathow focused on dynamic MRI. He proved significant correlations among diaphragm length and spirometric values vital capacity (VC), forced expiratory volume (FEV1) and other lung parameters [28].

### Relation of structural spine findings and LBP

The causes of LBP and their relations to spinal findings have been the subject of several studies, and continue to be a significant study topic. Harris [29] examined intervertebral discs from 123 subjects concluding comprehensiveness of the objective. Jensen [30] assessed low back magnetic resonance imaging (MRI) with the goal of finding structural changes related to LBP. Jensen found no direct connection between certain types of structural changes and LBP. The only structural change related to pain was disk protrusion. Carragee [31] studied MRI findings of 200 subjects after a period of low LBP, and found no direct significant MRI finding related to low back pain.

The way in which the diaphragm is used for non-breathing purposes is affected by it's recruitment for respiration [32]. There is evidence that the presence of respiratory disease is a stronger predictor for low back pain than other established factors [33]. However, the relationship between the respiratory function and the postural function is widely disregarded [34]. Body muscles coordination for posture stabilization is a complex issue, and the role of the diaphragm in this cooperation has not been intensively studied [35].

In this paper we presents an assessment of a non-respiratory diaphragm function via visual monitoring provided by magnetic resonance imaging (MRI). The main goal is to separate respiratory diaphragm movements from non-respiratory diaphragm movements, and then to evaluate their role in body stabilization. The subjects included in the study consisted of a group of healthy volunteers and a group of volunteers in whom structural spine disorders had been identified, and who suffered from chronic low back pain (lasting for one year at least). To the best of our knowledge, there has been no similar work dedicated to the postural function of the diaphragm in pathological cases.

We investigated diaphragm reactability and movement during tidal breathing and breathing while a load was applied to the lower limbs. We used diaphragm movement harmonicity, frequency and range for both respiratory and postural movements as assessed parameters. Another part of the parameters was acquired from static measurements, where we assessed diaphragm inclination, height and position in the trunk. Differences between healthy and pathological subjects were evaluated statistically. The results of our work should help in understanding the diaphragm function in the posture stabilization system, with possible implications for physiological practice.

## Materials and Methods

### 1.1 Subjects groups

Two groups of volunteers were selected:

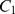
 — without a pathological condition (n = 16, 11 women, 5 men, control group), id numbers 1–16.

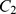
 — with a structural pathological condition of the spine localized in the lumbar spine area (n = 17, 8 women, 9 men, pathological group), id numbers 17–33.

Neither the healthy subjects nor the pathological subjects had any pulmonary disease. The average age of the control group was 35 years (in the 23–56 age span). The average age in the pathological subjects was 42 years (in the 23–65 age span). Detailed characteristics of the two groups are summarized in Table 1.

**Table 1:** Details of the study groups (mean 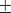 standard deviation).

	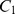	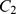
Age	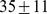	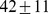
Weight (kg)	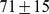	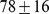
Height (cm)	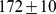	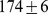
Sternum height (cm)	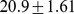	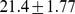
Thorax height (cm)	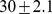	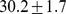

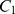
 is the control group, 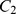 is the pathological group.

Structural findings in the pathological subjects were confirmed by the previous MRI in the lumbar spine area. The study excluded patients with an inborn defect of the spine or a defect acquired traumatically. All pathological subjects had suffered from low back pain of various intensity and frequency for at least one year (classifying the LBP as chronic). Types of the spinal pathologies are presented in Tab 2. The intensity of the LBP was determined using the visual analog pain scale (VAS) with a range of 0–10. The subjects indicated their current pain on the day of imaging and their overall pain in the course of one month before imaging. Length and frequency of the pain symptoms are shown in Table 2. The resulting scores are shown in Table 2. The VAS values for the control group 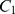 were zeros for all subjects.

**Table 2:** Pathological subjects' low back pain intensity, pain location and duration.

Subject id	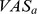	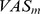	Pain frequency	Pain loc.	LBP duration (years)
17	1.1	6.2	twice a week	Cp, Lp	4
18	5.9	6.6	continuous	Cp, Lp	3
18	0	0	once or twice a year	Lp	20
20	0	2.4	twice a week	Lp	1
21	7.1	1.9	continuous	Lp	10
22	0.9	0.9	once or twice a mont	Lp	27
23	0.5	0.1	once or twice a year	Lp	2
24	5.1	3.1	continuous	Lp, Thp	4
25	6.3	7.1	continuous	Cp, Lp	22
26	5.3	5.3	continuous	Lp	1
27	0.2	6.4	once or twice a month	Cp,Lp	5
28	6.2	2.8	once or twice a year	Lp	16
29	2.2	4.9	once a month	Cp, Lp	9
30	6.6	8.9	once or twice a year	Lp	7
31	0	5.4	four times a month	Cp,Lp	1
32	2	3.7	continuous	Lp	5
33	0	7.7	continuous	Cp,Lp	30

Low back pain intensity values of the pathological subjects determined by visual analog scale (VAS for group 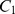 was all zeros). 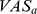 represents the actual pain felt on the day when the subject was measured. 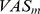 represents the pain felt in a period of one month before the measurements. All subjects exhibited different frequency of pain occurence and duration of the symptoms.

Acute pain was not the criteria for selection of the pathological group. The main criteria was the spinal findings, which is documented in Table 3.

**Table 3:** Pathological subjects' spine findings.

Subject id	Spine pathology
17	canal stenosis
18	disc protrusion
18	disc prolapse
20	disc degeneration
21	spondylolysis L5/S1, disc protrusion L5/S1
22	disc protrusion L1/L2, L2/L3
23	disc degeneration L4/L5
24	canal stenosis
25	canal stenosis
26	disc degeneration L4/L5 a L5/S1
27	disc degeneration L4/L5, end plates degeneration Th11/12
28	canal stenosis, end plates degeneration Th11/12 a Th12/L1
29	spinal canal stenosis, disc degeneration L4/L5 and L5/S1
30	spine stenosis, disc degeneration L4/L5 with protrusion
31	disc degeneration L4/L5 a L5/S1 with protrusion
32	disc protrusion L1/L2, L2/L3, canal stenosis, lig. flava hypertrophy
33	disc prolapse C5/C6

Pathological spinal condition observed during MRI spinal examination.

Due to a distinct inter-group difference in age, a paired t-test was performed to confirm no statistical significance among the groups. The resulting p-value (p = 0.08) showed no statistical difference at the 5% significance level. Normality of the age distribution within the groups by the Kolmogorov-Smirnov test (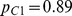, 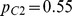) confirmed normal distribution of the data. No differences in the mean for all other parameters in Table 1 were confirmed with great significance (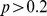).

### 1.2 Study settings

Diaphragm activity was monitored under two different situations:

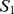
 — subjects lie supine on their backs during tidal breathing.

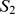
 — subjects lie supine on their backs during tidal breathing while loading is applied to the distal part of their extended lower extremities against the flexion of the hips. The applied pressure was of the 4th grade according to Janda's muscle test [36]. The subjects ensured that no additional pain was induced by the maneuver.

### 1.3 Ethics Statement

All patients provided written, informed consent for participation in the study and the study was approved by the Ethics Committee of the Motol University Hospital in Prague, Czech Republic.

### 1.4 Data Acquisition

The healthy group was examined in an open Siemens MRI apparatus, with a 0.23 T magnet and the NUMARIS/4 syngo MRI 2004A software load version. The length of each recorded sequence was 18 s. During this time, 77 images were recorded at regular intervals. The subject was in a supine position, using a size large body coil. The projection plane was placed sagittally in the axial topogram directed paravertebrally on the right side, midway through the center of the vertebral body and the edge of the thoracic wall. The width of each layer was 33 mm. The true FISP dynamic sequence was used, configured as follows: 1 NSA, matrix 240×256 pixels, TR 4.48 ms, TE 2.24 ms, FA 90, TSE1, FOV 328 mm.

The pathological study group was scanned by General Electric SIGNA HDx MRI, with a 1.5 T magnet and the 14-M5A software load version. The length of each recorded sequence was 22.2 s, resulting in the acquisition of 60 images. The projection plane was placed sagittally in the axial topogram directed paravertebrally on the right side, midway through the center of the vertebral body and the edge of the thoracic wall. The width of each layer was 15 mm. The GE FIESTA Cine dynamic sequence was used, configured as follows: 1 NSA, matrix 256×256 pixels, TR 3.1 ms, TE 1.3 ms, FA 55, FOV 420 mm.

**Figure 1:**
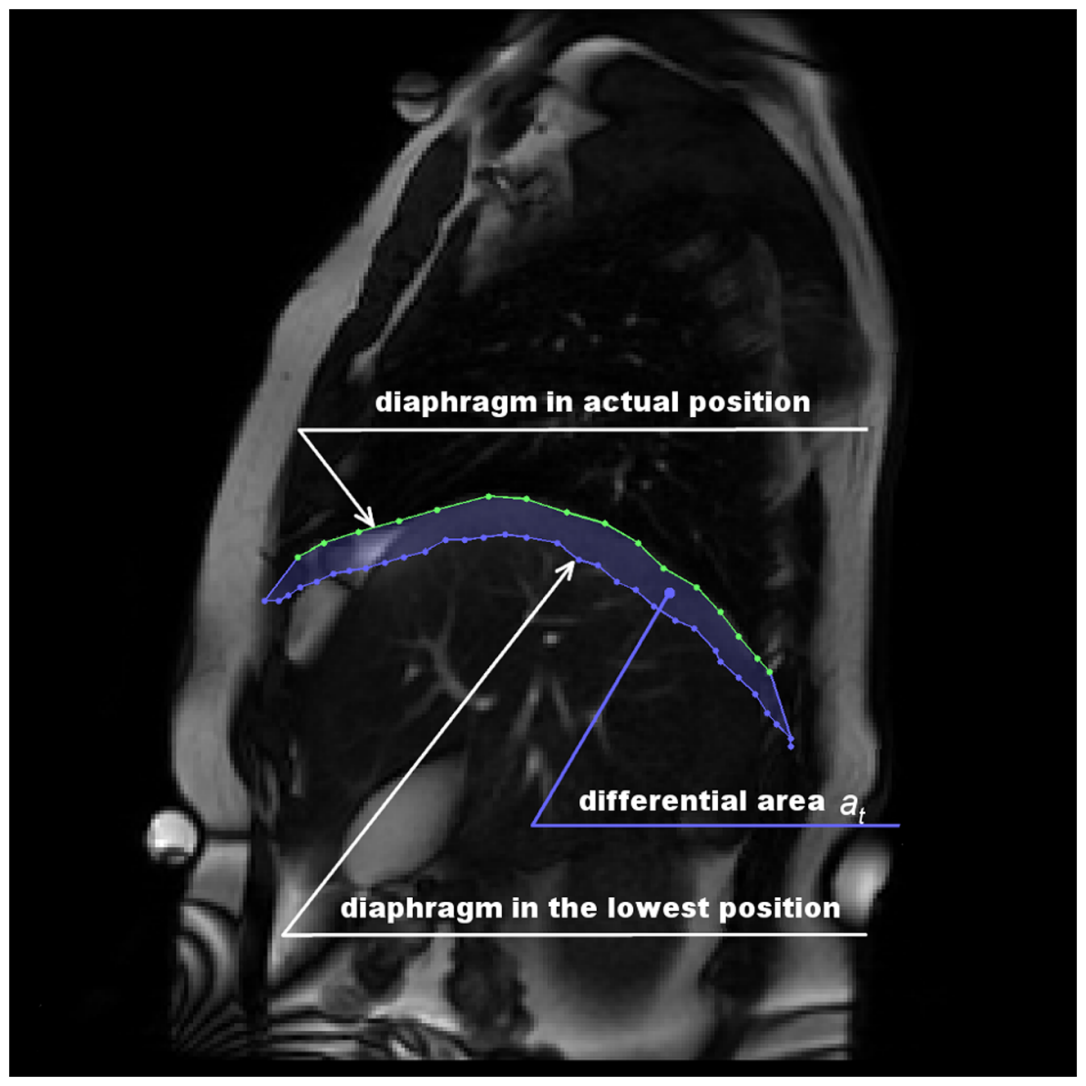
Differential area definition. Figure shows t-th image from a sequence with corrensponding diaphragm contour. The t-th diaphragm contour together with the lowest placed diaphragm contour in the sequence form the diferential area 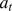.

The proposed processing methodology is indifferent to distinct images resolution, e.g. the resolution of the control group: 1.37 mm/pixel, pathological group: 1.64 mm/pixel. Three markers, 20-ml syringes filled with water, were placed on the skin surface of each subject on his right side. They are shown as hyper signal marks on the body surface (see for example Figure 1). The first marker was placed in the mid-clavicular line at the level of the jugular notch, and the second marker was placed at the level of the inferior 10-rib costal margin. The last marker was placed on the back of the subject at the level of the thoracolumbar junction.

The spatial resolution of our images was sufficient for proper diaphragm contour recognition on each sequence. In addition, the temporal resolution was sufficient, with a maximum recorded breathing frequency of 0.54 Hz. The diaphragm contour areas were not affected by artifacts. Image brightness was the only varying parameter, and there was no significant effect on the resulting differential curves.

### 1.5 MRI Processing

**Figure 2:**
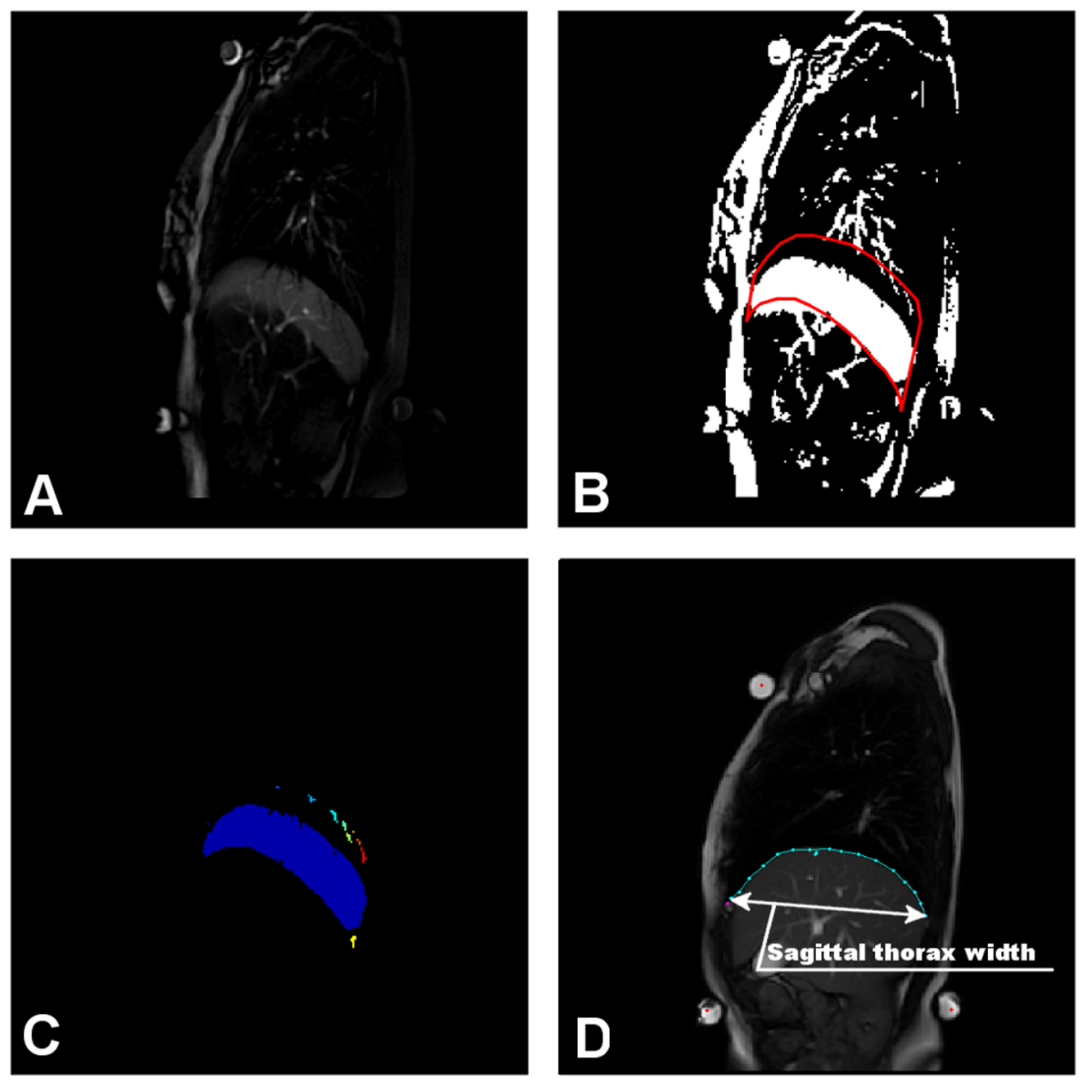
Differential area calculation. Image on t-th position in a sequence is subtracted from the background image (the image with the lowest placed diaphragm) (A). Subtracted image is thresholded, providing a binary image with a clearly visible crescent corresponding to movement of the diaphragm (B). The red-bordered part, surrounding the highest and the lowest diaphragm position from the whole sequence reducing the space for crescent location. Continuous image parts inside the border are labeled and the part corresponding to diaphragm movement is than processed (C). Some of the extracted parameters were normalized using the thorax width measure shown here (D).

**Figure 3:**
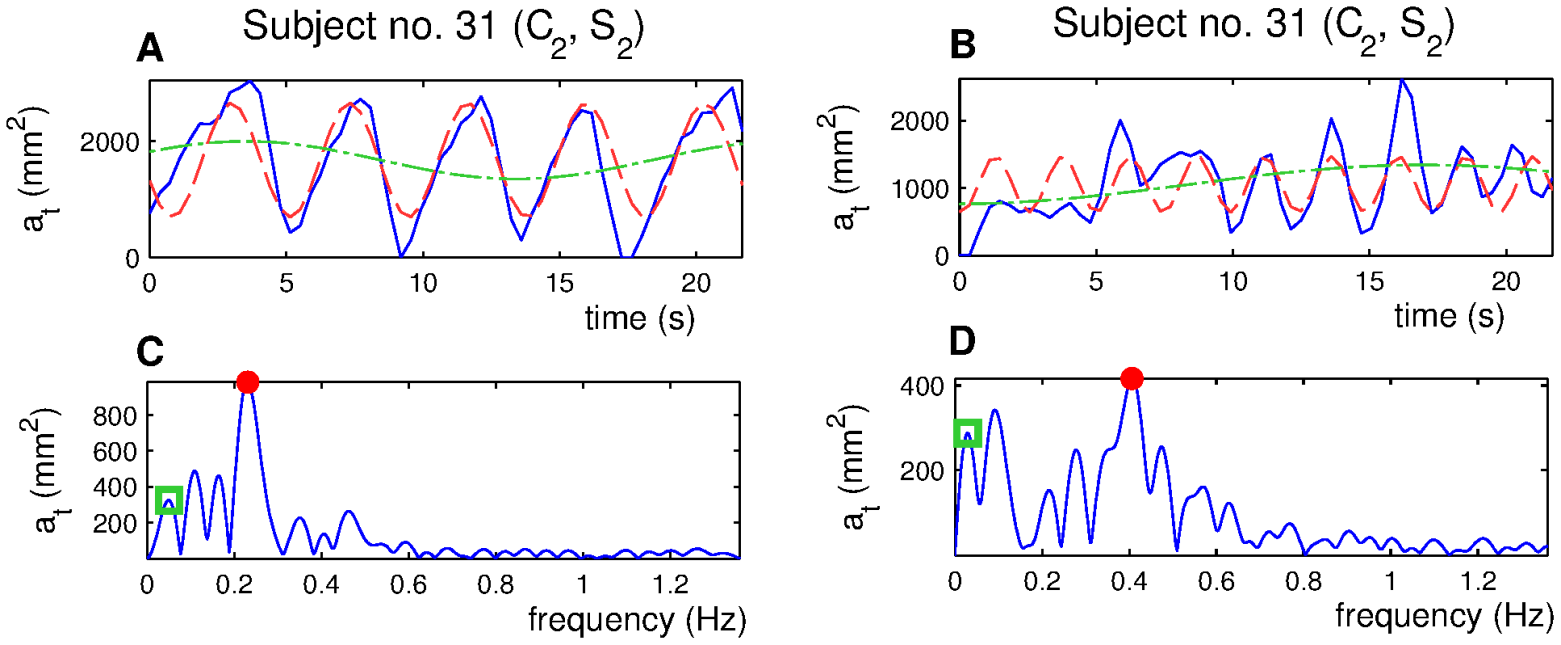
Dif-curves (A, B, solid line) and appropriate spectra (C, D, solid line). Extracted res-curves (red dashed line, A, B) and pos-curves (green dotted line, A, B) with corresponding spectral peaks (C, D) marked in the spectra with a red dot (respiratory peak) and a green square (postural peak).

**Figure 4:**
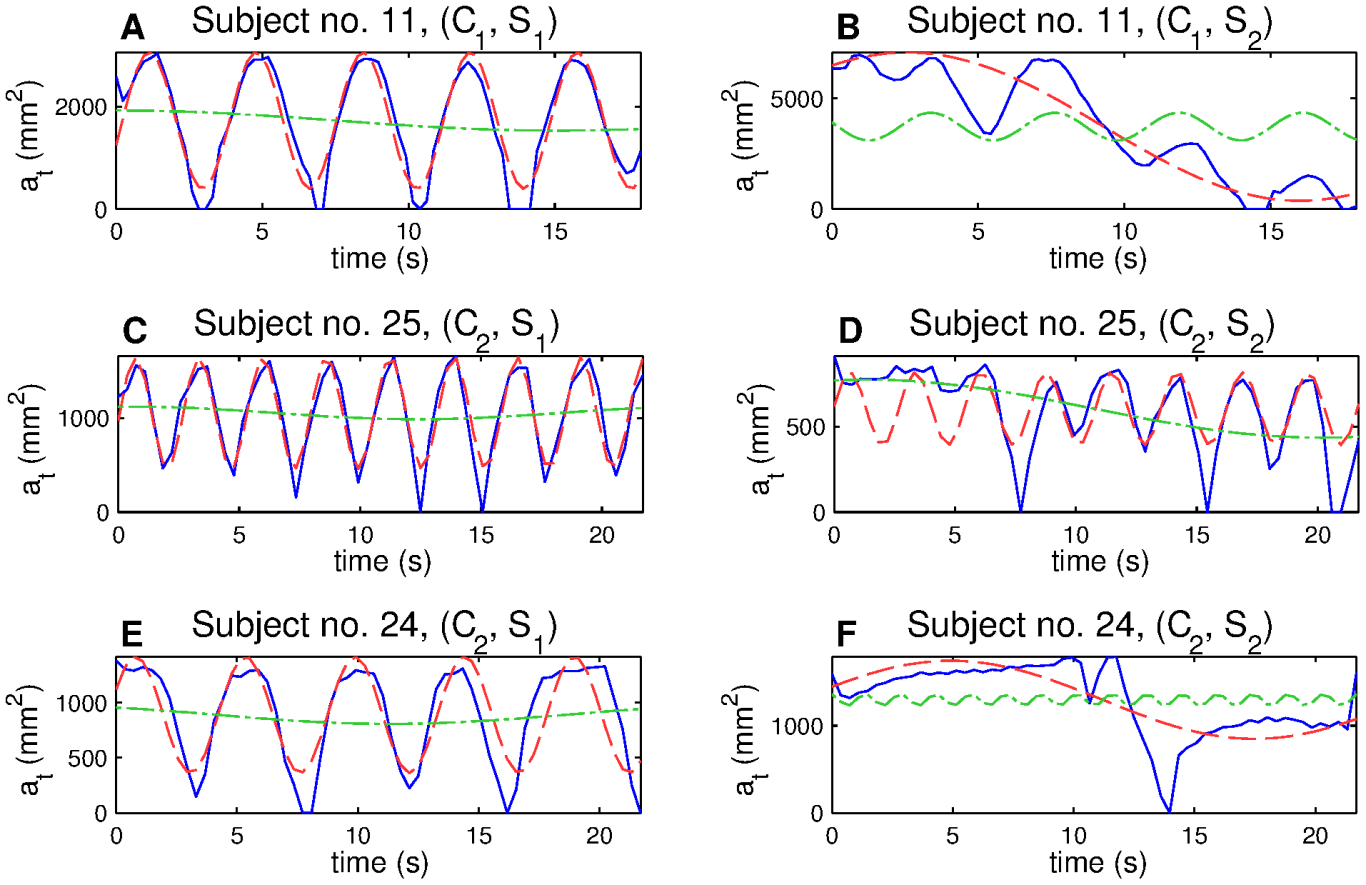
Dif-curves (solid line) and extracted res-curves (red dashed line) and pos-curves (green dotted line). Example of harmonic breathing (A), breath with a strong postural part after the load occurred (B), harmonic breath which became partly non-harmonic after the load occurred (C, D), and breath which almost lost its ability of respiration movement ability after the load occurred (E, F).

In order to assess diaphragm activity, the **differential curve** (dif-curve) was calculated across all MR images. Firstly, let us define the **differential area (** 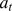 **)** as the area bordered by the diaphragm in the lowest position from the sequence and the diaphragm in current (t-th) image — see Figure 1. The image containing the lowest placed diaphragm is called the background picture. Secondly, the dif-curve is defined as the time series of 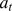, measured in 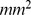. Hence, the dif-curve is an integral quantity which characterizes the diaphragm motion in the same manner as spirometry, but it consists strictly of diaphragm movement. The algorithm for 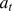 calculation is shown in Figure 2A–C. Typical dif-curves are shown in Figures 3, 4.

### 1.6 Extraction of respiratory and postural movements from the dif-curve

Each digitally sampled signal can be expressed as the sum of a finite number of harmonic waves of different amplitudes and phases. Decomposition of the signal into harmonic components is traditionally represented by the harmonic spectrum of the signal. The spectrum denotes the relation between the amplitudes and the frequencies of the harmonic waves. Typical dif-curves spectra are shown in Figure 3. Each peak in the spectrum stands for one harmonic component. If the diaphragm worked only for respiration with stable depth of the motion it would lead to a simple spectrum with a single peak corresponding to the breathing frequency. This motion would be fully described by a single sine wave. Diaphragm motion is more complex, and often involves other non-respiratory movements. However, due to the harmonic properties of respiration, the harmonic spectrum is useful for dif-curve processing.

We chose to model diaphragm motion by two sine waves corresponding to respiratory and non-respiratory movements. These waves are extracted from the dif-curve spectrum by the inverse Fourier transform. The two models are shown in Figure 3A,B. The original dif-curve is plotted by a solid line. The respiratory model is called a **respiratory curve (res-curve)**. The non-respiratory model is called a **postural curve (pos-curve)**. The res-curve fully characterizes the respiratory movement by frequency and amplitude. The pos-curve provides a model of a postural global range by a pos-curve amplitude. If there were several peaks that together compose the final respiratory or postural part of the signal, we chose by visual inspection the peak that best described the values of the original signal (peaks and subsequent pos-curves are marked in Figure 3 by a green square).

The relation between breath regularity and the spectrum complexity of the corresponding dif-curves is summarized in Fig. 3. Fig. 3A,C provides an example of a dif-curve with the corresponding spectrum for a person whose respiratory movements are widely regular. In the corresponding spectrum there is one significant peak, which represents the subject's respiration marked by a red dot. The diaphragm respiratory movement is also modulated by other movements. This causes the occurrence of smaller peaks besides the respiratory peak. These peaks capture smaller parts of the diaphragm movement. Fig. 3B,D shows a more complex dif-curve with less regular respiration. The spectrum (Fig. 3D) has a clearly visible peak, which corresponds to the respiration (marked by a red dot) and, again at lower frequencies, there are peaks which modulate the respiratory movement. This time, however, the peaks capture a much bigger part of the diaphragm movement.

Three typical dif-curves with corresponding respiratory and postural models for both situations, 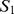 and 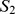, are shown in Figure 4. There is a clearly visible respiratory function (A) with a big postural movement in situation 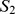 (B) for subject 11. Subject 25 (in E, F) did not respire for the first six seconds of the imaging in situation 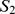, and then the respiration became regular. The subject 24 (C, D) exhibited almost no respiration during situation 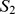. Dif-curves including no respiration led to exclusion of the subject from further statistical data processing.

### 1.7 MR Parameters Extraction

Two sets of parameters were extracted on the basis of diaphragm MRI activity: **Dynamic parameters** are based on dif-curve processing. The main aim of introducing dynamic parameters was to assess which part of diaphragm motion is related to respiration, and how significant non-respiratory movements are.

**Figure 5:**
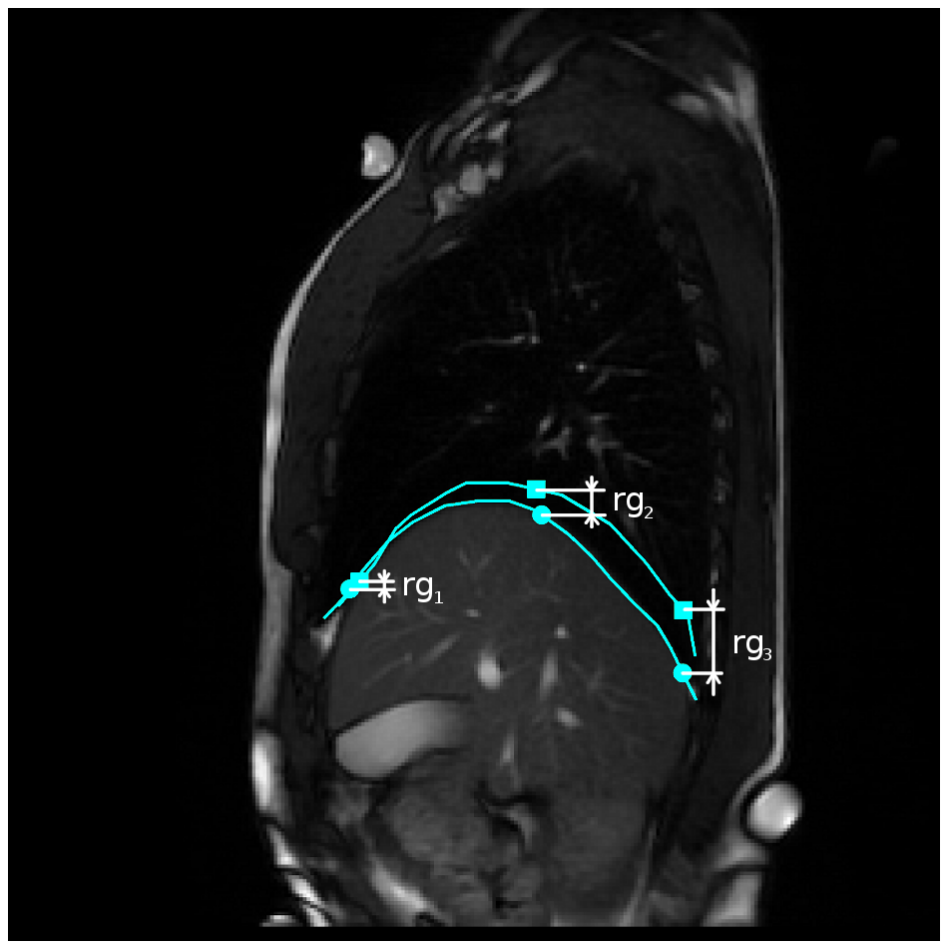
Parameters 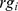. Parameters 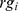 are computed as vertical subtraction of caudal from cranial diaphragm position. The three parameters correspond to the anterior (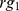), middle (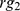) and posterior (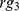) diaphragm part. Points were spread evenly on the diaphragm contour with small constant drift of 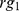 and 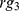 from the contour margins.

- Frequency and amplitude of res-curve: 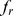, respectively 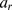.
- Amplitude of pos-curve: 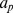.
- Amplitudes ratio of res-curve and pos-curve: 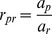.
- Range of diaphragm motion measured in 3 different points placed on diaphragm surface 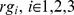. See Figure 5. Measured in mm.
- The percentage of energy yielded by the three biggest spectrum lines: 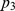.
- Standard deviation, skewness and kurtosis of the dif-curve: 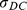, 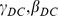.

**Figure 6:**
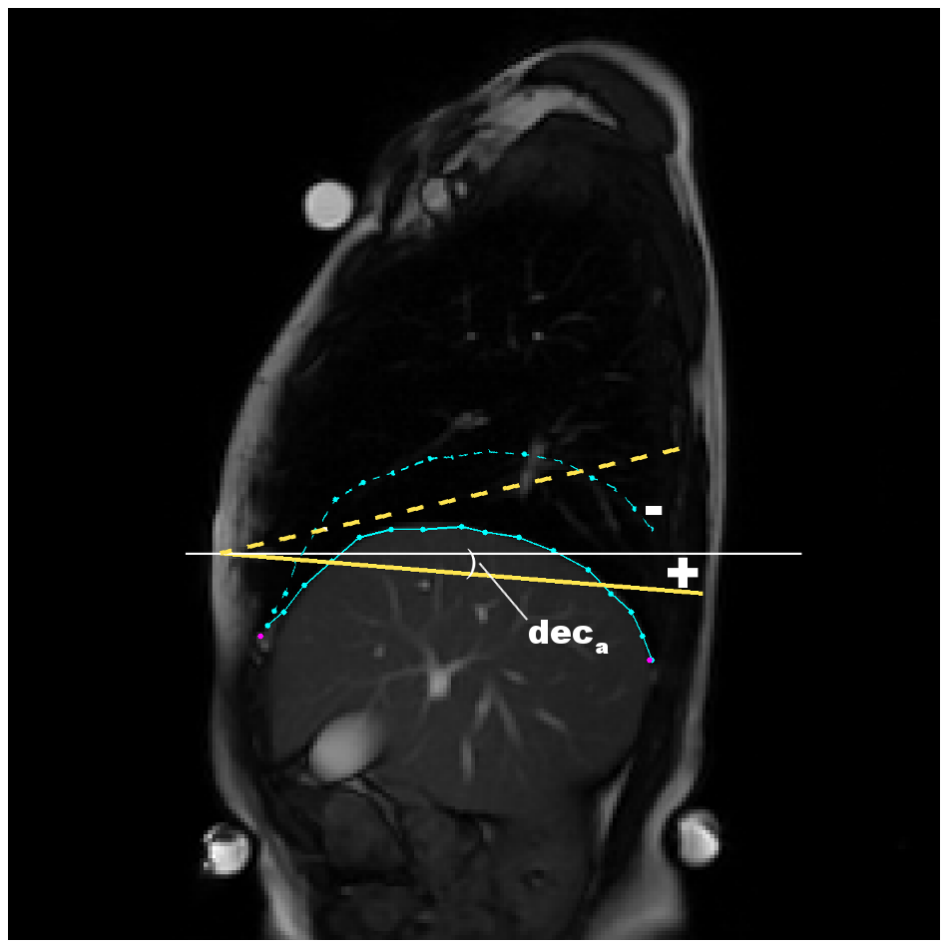
Measurement of the diaphragm inclination. Inclination of the diaphragm was measured by angle between the line fitted to the diaphragm contour and horizontal axis. The inclination was measured during the caudal diaphragm position.

**Figure 7:**
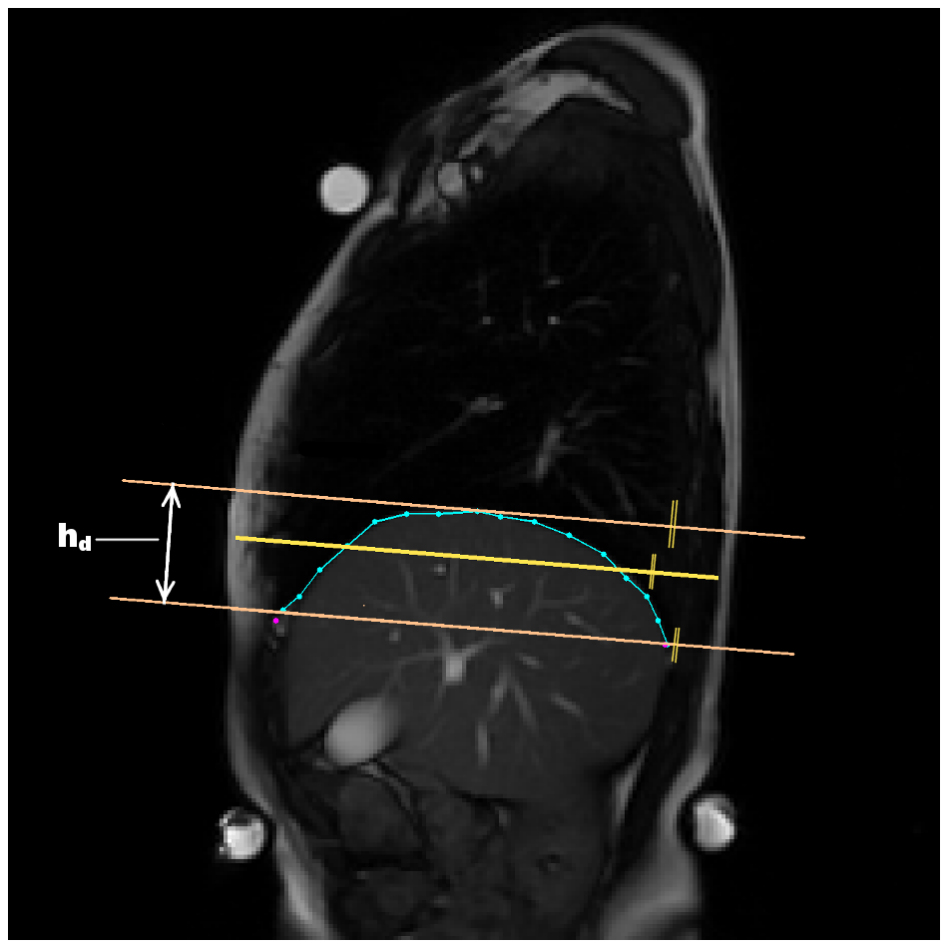
Measurement of the diaphragm height 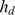. Measurement was done during the diaphragm caudal position. The middle line is the line fitted to the diaphragm contour by least squares method.

**Figure 8:**
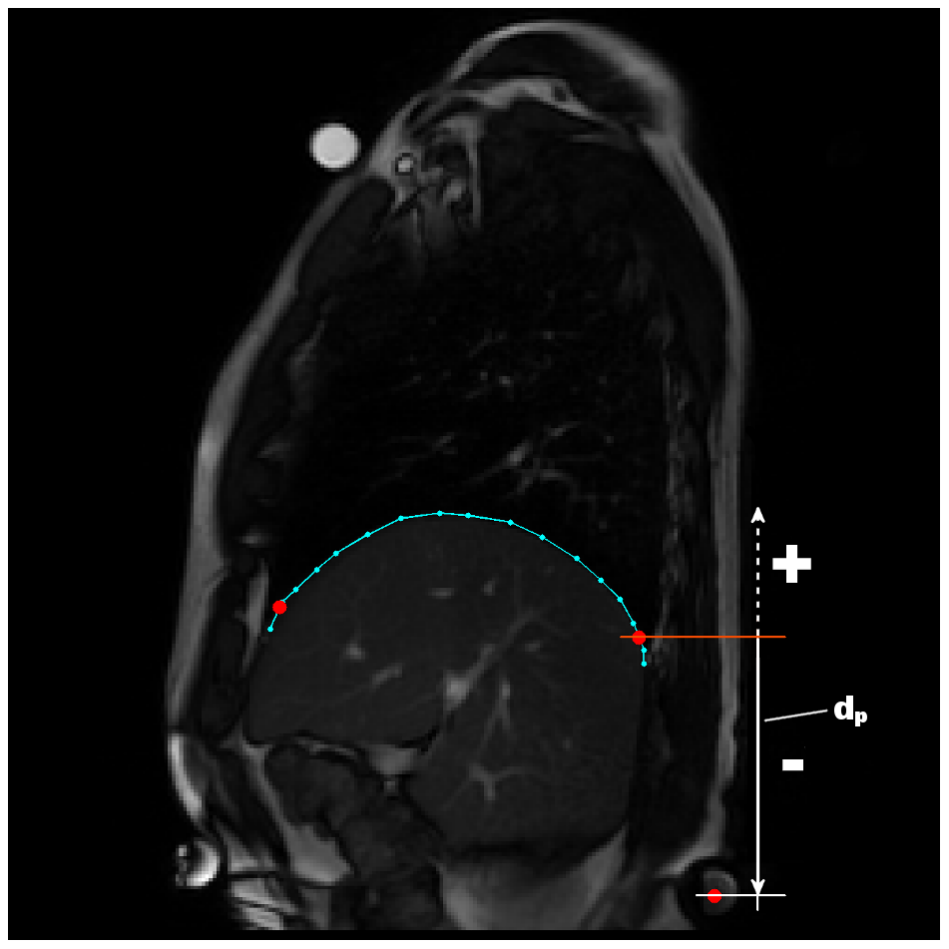
Measurement of the diaphragm height in the trunk. The figure indicates when the height is negative (the diaphragm higher than the back marker) and when the height is positive (the diaphragm placed under the back marker).

**Static parameters** assess diaphragm shape and position. For the static parameters, the following features were extracted in order to analyze the anatomic characteristics of the diaphragm:

- Diaphragm inclination in the sagittal plane in caudal position: 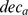. Angle measured as shown in Figure 6.
- Height of a strip overlapping the diaphragm contour parallel diaphragm inclination: 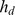 — see Figure 7.
- Vertical distance from anterior point used for 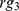 and back marker (syringe): 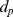. This parameter corresponds to the diaphragm height in the thorax. See Figure 8.

### 1.8 Statistical Analysis

A paired t-test was used to identify differences between the control and the pathological group. The significance of the statistical test is marked by symbol 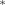, or by symbol 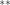 if level of significance was below 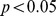, respectively 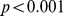, as indicated in Tables 4, 5, 6 in Section 2. A Kolmogorov-Smirnov (KS) analysis was performed, to assess the normality of the data.

**Table 4:** Dynamic parameters results, first part.

		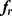 ( 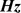 )	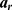 ( 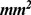 )	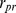 ( 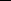 )	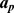 ( 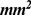 )	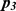 ( 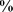 )
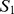	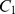	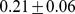	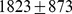	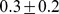	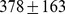	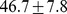
	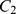	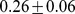	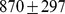	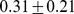	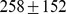	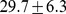
	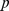	*	**	–	*	**
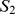	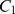	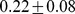	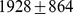	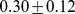	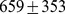	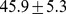
	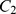	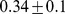	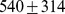	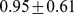	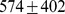	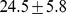
	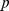	*	**	*	–	**
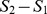	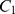	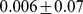	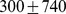	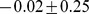	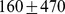	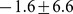
	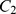	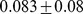	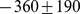	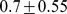	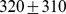	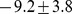
	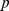	*	*	**	–	*

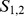
 stands for the monitoring situations, 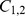 stands for the subjects groups, 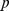 stands for p-value of the student's t-test among the groups. 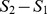 stands for subtraction of parameter — assess change of the parameter after a load was applied to the lower limbs. The parameters are: frequency (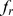) and amplitude (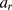) of res-curve. Amplitude of pos-curve (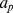). Amplitudes ratio of res-curve and pos-curve (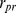). The percentage of energy yielded by the three biggest spectrum lines (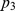).

**Table 5:** Dynamic parameters results, second part.

		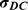	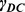	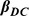	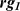 ( 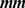 )	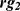 ( 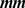 )	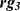 ( 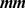 )
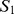	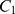	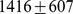	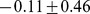	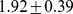	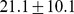	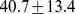	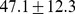
	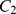	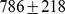	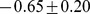	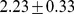	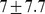	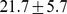	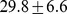
	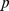	**	**	*	**	**	**
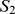	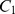	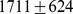	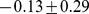	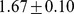	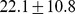	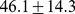	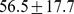
	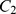	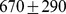	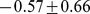	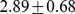	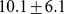	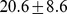	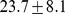
	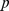	**	*	**	**	**	**
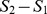	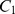	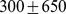	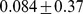	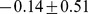	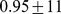	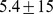	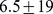
	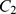	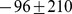	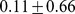	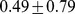	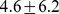	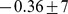	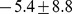
	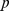	*	–	*	–	–	*

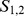
 stands for the monitoring situations, 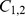 stands for the subjects groups, 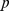 stands for p-value of the student's t-test among the groups. 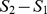 stands for subtraction of parameter — assess change of the parameter after a load was applied to the lower limbs. The parameters are: standard deviation (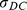), skewness (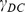) and kurtosis (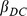) of the dif-curve. Range of diaphragm motion measured in 3 different points placed on diaphragm surface 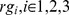.

**Table 6:** Static parameters results.

		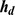 ( 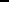 )	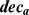 (  )	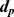 ( 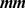 )
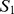	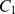	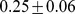	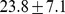	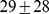
	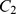	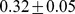	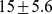	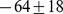
	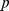	*	**	**
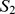	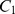	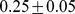	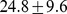	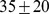
	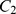	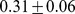	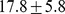	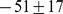
	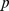	*	*	**
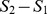	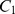	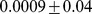	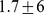	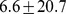
	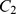	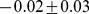	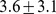	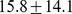
	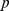	–	–	–

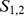
 stands for the monitoring situations, 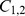 stands for the subjects groups, 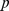 stands for p-value of the student's t-test among the groups. 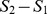 stands for subtraction of parameter — assess change of the parameter after a load was applied to the lower limbs. The parameters are: diaphragm inclination in the sagittal plane in caudal position (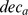). Height of a strip overlapping the diaphragm contour (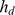). The diaphragm height in the thorax (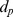).

The correlation (by Pearson's correlation coefficient) between all parameters and the subjects body mass index (BMI) was assessed in order to eliminate an effect on the results. The parameters affected by BMI dependence were in situation 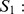 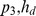 and in situation 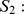 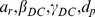. A possible way to suppress the correlation with BMI was to normalize the parameters by the width of the subject's thorax (Figure 2E, the width was determined during the lowest position of the diaphragm). However, no influence on the statistical results was observed after normalization, except for parameter 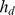, which t-test results changed by two orders of magnitude (though there was no change in significance). In order to keep results clear, all were kept in the original units, with the exception of 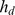, which is in normalized form.

All extracted features were treated for outlier values. Outlier values were determined as follows:
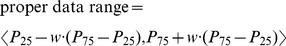
(1)

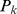
 stands for k-th percentile, 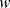 is a constant set by default to 1.5. This value ensures approximately 99.3 percent coverage of the data, when the data is normally distributed. Data outside this range is likely to consist of error values or marginal data that distorts the statistics.

Secondly, as stated in the methodology section (Sec. 1.6), patients were present in our datasets whose respiration did not exhibit proper respiration movement. These subjects were also excluded from the statistical evaluation — four subjects, all from the pathological group 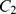 (id numbers: 19, 24, 27, 29).

## Results

The results are presented in Tables 4, 5, 6.

### 2.1 Dynamic parameters

#### 2.1.1 Respiratory and postural curves

We concluded significantly faster respiration in pathological group in both observed situations 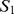, 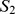, with 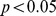. Respiratory frequency did not change much for the control group after a load was applied to the lower limbs (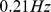 in 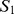, 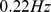 in 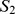). By contrast, the frequency of the pathological group rose significantly (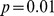). The height of the diaphragm respiratory movements reflected by the respiratory curve amplitude (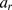) resulted in very significant difference among the groups, with 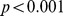 in both situations 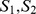. As in the case of respiratory frequency, there was no change in respiratory curve amplitude in the control group when a load was applied to the lower limbs (1823 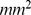 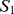, 1928 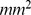 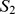). By contrast, the pathological group showed lowered excursions when load was applied (870 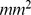 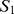, 540 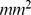 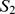). The inter-situational difference was significantly different amongst the groups with 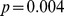. In comparison with the pathological group, the control group had 3 times bigger excursions in situation 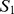, and 6.5 times bigger excursions in the situation 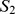.

In order to compare diaphragm excursions in mm, 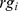 parameters were introduced. The diaphragms excursions was measured in three points laid on the diaphragm contour — anterior, central and posterior part (see Figure 5). The control group exhibited a significantly bigger motion range than the pathological group in both situations (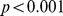). In addition, the measurements showed great motion of the posterior diaphragm part than of the anterior part. In 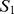, the antero-posterior ratio was 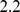 within the control group and 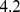 within the pathological group. In 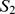, the control group raised the range of the posterior part to 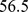 mm, resulting in an antero-posterior ratio of 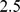. The pathological group, by contrast, raised the range in the anterior area and reduced the range in posterior area, resulting in an antero-posterior ratio of 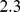.

The range of postural movements (the amplitude of the postural curve 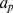) was great in the control group (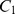: 380 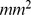 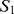, 660 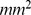 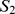, 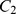: 260 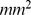 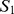, 570 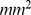 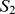), with the only statistically significant difference in situation 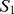 (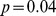). For both groups, the amplitude of the postural curve rose when a load was applied to the lower limbs, while the rises in 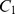 and 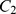 did not differ significantly (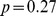). The amplitude ratio of the res-curve and the pos-curve 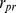 shows which type of diaphragm motion dominates in the overall motion. When this parameter is greater than 1, it means that postural moves of the diaphragm are bigger than the respiratory moves, and vice versa. Moreover, in situation 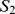 the range of motion in the pathological group was equally distributed between respiratory and postural movement ranges (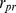 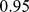, meaning 50% of the total motion range by postural motion and 50% by respiratory motion), while both in situation 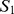 and in situation 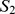 the control group had the same ratio of postural and respiratory movements of 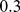 (23% of the total motion range for postural motion and 77% for respiratory motion).

#### 2.1.2 Diaphragm motion harmonicity and central moments

The most important dif-curve shape parameter is its harmonicity, reflected by parameter 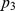. When the patient loses control over the diaphragm motion, the dif-curve loses its typical harmonic shape and the three biggest spectral lines carry less of the signal energy (see Section 1.6). The control group was able to keep the harmonicity almost at the same level in both situations (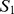 46.7%, 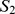 46%), while the pathological group achieved a significantly (p-values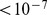) lower percentage (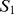 29.7%, 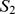 25.5%). For the pathological group, the decrease in the 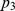 value was significantly bigger (p-value 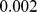) bigger than decrease for the control group.

The third central statistical moment, skewness (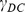), elegantly characterizes the centering of the dif-curve around it's mean value. This parameter can be used to indicate whether the patient kept the diaphragm longer in inspiratory position or in expiratory position. Naturally, harmonic breath would lead to zero skewness. If the diaphragm is kept longer in inspiratory (caudal) position there is positive skewness, and if the diaphragm is kept in longer in respiratory (cranial) position there is negative skewness. In 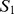, both the control and the pathological group had negative skewness (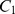: 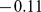, 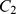: 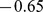). However, the control group exhibited big variance (positive skewness in a case of 6 subjects). For the pathological group, all values were negative except in the case of one subject. The difference is significant (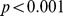), despite big variance in the control group. In 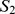, the mean skewness values were 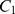: 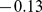, 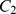: 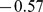. The control group became more consistent, while the pathological group exhibited great variance for this parameter. This is due to an increase in the influence of the postural part of the diaphragm movement. The difference between groups 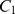, 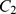 was significant, with 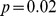. There was no significant change, either in the control or in pathological group, when a load was applied to the lower limbs (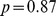).

The fourth central statistical moment kurtosis can be used to study control over the diaphragm movement. Harmonic motion shows lower kurtosis than more random, worse controlled motion. In situation 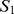, the control group had a lower kurtosis parameter (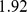) than pathological group (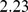), with a significant difference, 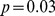. In situation 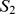, the kurtosis parameter for the control group fell to 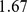, and for the pathological rose to 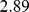, which raised the significance of the inter-group difference (p-value = 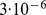).

### 2.2 Static parameters

The diaphragm height, described by the 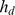 parameter (higher 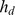 means a more bulging diaphragm), differs significantly between the groups, both in situation 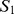 (p-value = 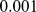) and in situation 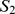 (p-value = 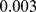). The parameter was independent of postural load, and has a very similar value for both situation 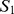 and situation 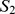: 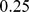 for the control group, and 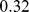 for the pathological group. The parameter was normalized by the anteroposterior size of the thorax. In addition, the dependency of the parameter on the patient's pain intensity was revealed (see Section 2.4).

The inclination of the diaphragm in caudal position (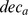) differs significantly between the groups in the two observed situations (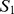, 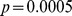, 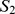, 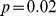). The difference between situation 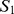 and situation 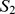 was not great (within the standard deviation range), and was statistically the same for both groups (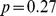). The mean inclination in situation 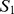 was 23.8 in the control group and 15 for the pathological group, i.e. the control group kept the diaphragm in a more vertical position.

The diaphragm height in the thorax (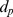) differs considerably between the groups 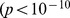). The control group kept the diaphragm below the back marker in both situations. In situation 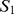 the value was 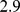 cm, and in situation 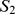 the value was 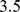 cm. The diaphragm was lowered by 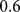 cm on an average, which is a small value in comparison with the standard deviation of the values. In situation 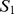 the pathological group had the diaphragm in a position 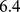 cm above the back marker, on an average, and 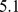 cm above the marker in situation 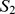. The average difference is 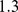 cm. No statistically significant difference (p-value 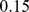) was found among the diaphragm shifts after a load was applied to the lower limbs.

### 2.3 Summary

We concluded that there was slower and deeper respiratory motion (parameters 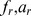) for both observed situations. In addition, after the postural demands rose in situation 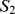, the breathing speed increased significantly (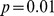) in the pathological group. In the same manner the breath depth (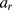) lessened significantly (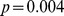) in the pathological group. There were bigger postural moves in the control group, and they remained bigger in both situations, rising equally for each group. The res/pos ratio 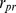 shows great domination of postural moves in the pathological group. As the respiratory moves lowered when there was a load, the ratio rose greatly in the pathological group, and the difference between the groups became significant. A very significant difference in harmonicity emerged, which is denoted by the 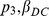 parameters. These parameters indicates a much more harmonic diaphragm movement in the control group, with or without a load. In addition, 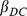 increased significantly (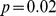) in both situations in the pathological group. The diaphragm motion in the thorax was symmetrical for the control group.

The results for the static parameters revealed that the diaphragm of the control group was flatter (parameter 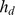) in both situations. The inclination of the diaphragm was greater (i.e. it was more verticalized) in the control group. The pathological group had the diaphragm placed significantly higher in the trunk, as indicated by the 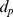 parameter.

### 2.4 Correlation between pain intensity (VAS), pain duration and the measured parameters

**Figure 9:**
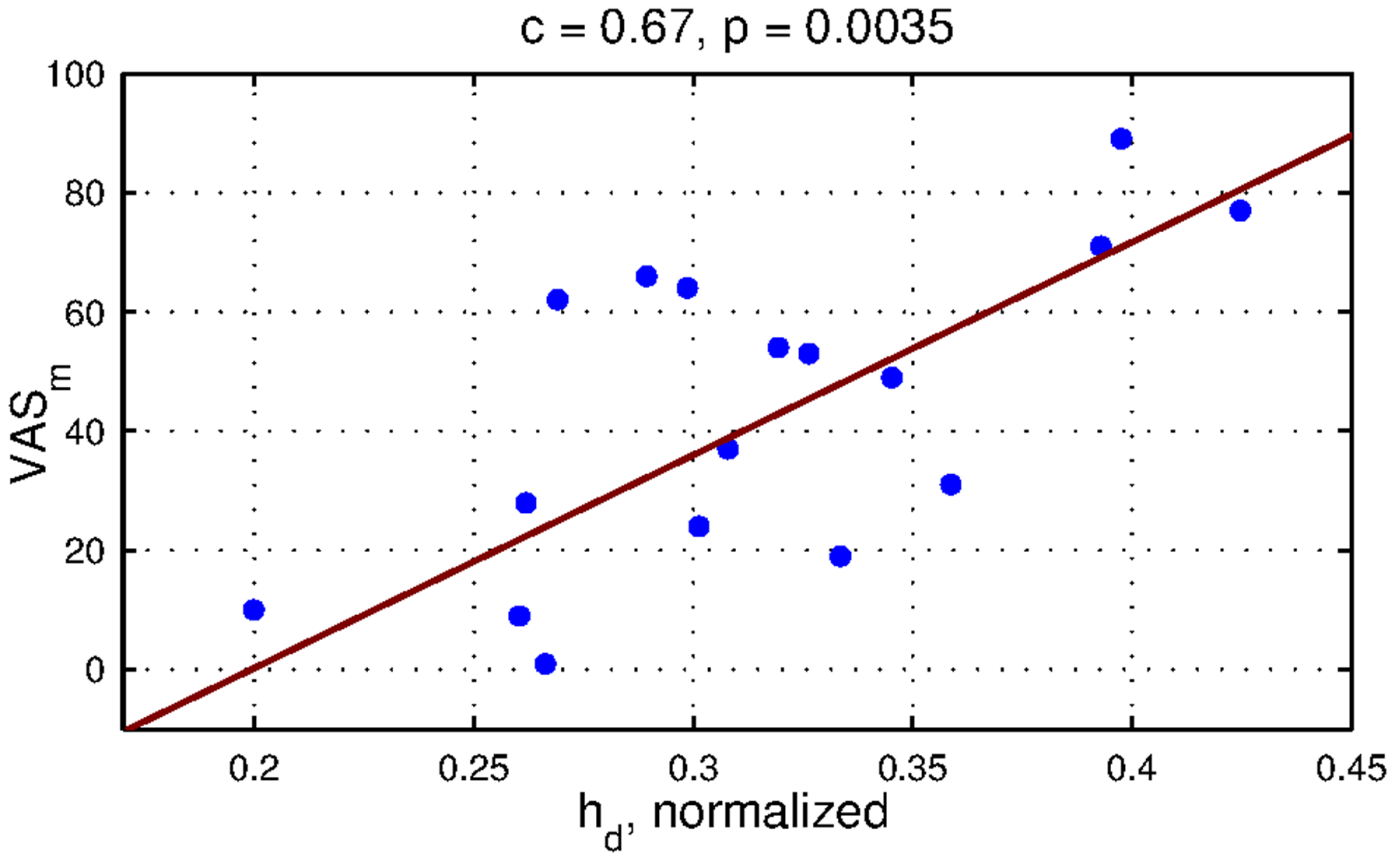
Correlation between 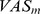 parameter and 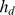 parameter in situation 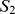. Diaphragm height were the only diaphragm parameter which was statistically significantly correlated (p = 0.0035) with the subjects' low back pain indicated during the month before imaging. Pearson correlation coefficient was 0.67.

A correlation analysis between VAS of the subjects' LBP intensity and the measured parameters revealed that the only correlated parameter was 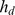 (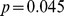). A significant correlation emerged only in situation 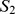 (Figure 9). The only significant correlation was detected for VAS summarized for the month before imaging. There was no significant correlation between diaphragm motion harmonicity or range and the intensity of the subjects' LBP. No correlation was detected between the parameters and pain duration either.

## Discussion

Studies of diaphragm motion using MRI are taken as a valid method for intrathoracic movement investigations [18]–[20]. Plathow [18] assessed diaphragm length using dynamic MRI in the mid-coronal plane by 1.5 T magnetic resonance, and concluded that the spatial and time resolution is sufficient for acquiring the breathing sequences. Gierada [20] also used a 1.5 T MRI device for measuring the height of the excursions of the diaphragm at three different points in several sagittal planes. Gierada [19] assessed MRI artifacts and concluded that MRI is a valid method for diaphragm image processing along the diaphragm contour. Suga [23] used breathing MRI (BMRI) for comparing healthy subjects and subjects with chronic obstructive pulmonary disease (COPD), measuring excursions and the length of the apposition of the diaphragm in supine position. Suga concluded that BMRI is a useful non-invasive method with good spatial and temporal resolution.

The extracted parameters were selected in a way that allows a wide spectrum of diaphragm properties to be assessed. A novel method involves evaluating harmonicity using statistical methods (kurtosis) or harmonic spectrum processing. Some similar parameters to ours can be found in the literature — measurements of cranio-caudal excursions of the diaphragm [23], [26], [37]–[39] and the anteroposterior and lateral proportion of the diaphragm [21], [22]. Plathow [40] measured shortening of the diaphragm contour in the sagittal and frontal plane. The height and anteroposterior proportion of the diaphragm were assessed in [10], [20], [24]. Miyamoto [41] assessed the curvature of the diaphragm. Differences between the diaphragm in inspiratory and expiratory positions, measured by Gierada [20] and Takazakura [42], were used to determine the height of the diaphragm motion. Gierada [19] compared the movement of the ventral and dorsal part of the diaphragm using MRI. Kolar [17] used measurements of the differential area.

In the results section, we concluded that there is a statistically significant difference in the range of motion (ROM) of the diaphragm. A two and three times greater ROM was noted in the control group, than in the pathological group in situations 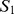 and 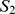. In addition, the average diaphragm excursions 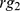 (central part) in situation 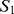 were 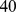 mm in the control group and 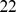 mm in the pathological group. In situation 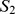, 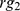 was 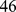 mm in the control group and 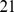 mm in the pathological group. The diaphragm excursions rose from the ventral part to the dorsal part. Gierada [20] also concluded that there was a bigger motion range in the ventral diaphragm of the diaphragm than in the dorsal part. Kondo, who studied the correlation between lung volume and diaphragm motion, came to the same conclusion in [17]. Kolar [39] observed diaphragm excursions 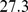 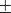 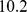 mm in the apex and 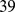 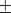 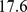 mm in the dorsal part during tidal breathing. Takazakura [42] showed a difference of 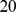 mm within the highest point of the diaphragm motion when sitting and when supine. Taking into account the large range of diaphragm motions reported in the literature [43], our measurements prove to be consistent.

When considering changes in the range of diaphragm motion after pressure was applied to the lower limbs, the ROM values for the control group rose on an average, but there was great variance in the group, and the rise was bigger in the posterior part than in the anterior part. The ROM values for the pathological group rose in the anterior part of the diaphragm, and lessened in the posterior diaphragm part. In contrast to our measurements, Kolar [44] observed an opposite change in the same situations. In Kolar's case, the range of motions was the same during tidal breathing, but the group with LBP had lower excursions of the anterior part of the diaphragm. The subjects in Kolar's study had the diaphragm at the same height in the trunk, despite the symptoms. We observed that the diaphragm was significantly higher for the pathological group. This may be a mechanism by which the pathological group was able to keep the diaphragm excursions more evenly spread after the postural demands increased.

We also observed that the diaphragm was more contracted in the posterior part for the control group. Diaphragm inclination measurements showed significant lowering of the posterior part of the diaphragm relative to the anterior part of the diaphragm for the control group. The pathological group kept the diaphragm in a more horizontal position. The average changes in inclination after a rise in postural demands were only small in comparison with the variance of the inclination. The height of the diaphragm contour (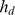) above the zone of apposition was also measured as a significant parameter between the groups of subjects. Suwatanapongched [43] concluded that there was flattening of the diaphragm in the older population in his study. Our results did not show any significant age-related correlation of diaphragm flatness. Instead, the only significant correlation that we observed was between diaphragm height and the LBP intensity of the pathological group during the month before the measurements were made. The correlation was significant in situation 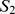. We assume that this diaphragm bulging is due to worse ability to contract the diaphragm properly. To the best of our knowledge, there are no results in the literature for measurements of diaphragm flatness in subjects suffering from LBP. Worse ability to contract the diaphragm in the pathological group is also supported by the significantly higher position in the trunk.

Other questions which emerge in relation to LBP intensity are the effects of acute pain. These would bias our findings, as the study focused on long time changes in the motion patterns of the diaphragm. The first factor is the pain induced by the applied load. This was controlled by our methodology, and the subjects ensured that no additional pain was induced by postural load. The second factor concerns differences in pain intensity perceived on the day of measurement, and the influence of the pain on the results. An important consideration is that the pain was chronic, and so we assume a tendency of the muscles to overload the spine, and some influence of the observed structural degenerative spinal findings. The range of pain intensity on the day of measurement of the patients is wide, from 0 to 8.9 (refer to Table 2). This wide range of pain intensity is useful for revealing a possible dependency of the parameters on acute perception of the pain. The best practice would be to classify the subjects into groups according to pain, and to treat the groups statistically. However, the kind of evaluation was not possible, because we would have needed many more study subjects. A second option was to examine the correlation between pain intensity and the measured parameters. No correlation was concluded between measured parameters and pain intensity except for bulging (i.e. long term pain) of the diaphragm, as was discussed above. The results indicate that, as the pain is long term, the patients do not change their respiratory patterns according to fluctuations in the chronic LBP.

It was concluded that a useful method for comparing the ratio of actual respiratory-related motions with other motions of the diaphragm is to separate the differential curve into respiratory and non-respiratory movements of the diaphragm. This division was inspired by various works. In [45]–[47] the postural and respiratory functions of the diaphragm were assessed using invasive EMG. Hodges [3], [46], [48] described tonic and respiratory activation during the breathing cycle and superimposition of the electromyographic signals phasically related to harmonic limb movement. Hodges also used the harmonic spectrum in his investigation of muscle cooperation for compensating breathing movements in body posture. Our study showed a non-negligible proportion of non-respiratory diaphragmatic motion, referred to as postural movements. These movements formed one third of the diaphragm motion range, on an average, in tidal breathing. The rise in the range of postural motions when there is an increase in postural demands on the body confirms the participation of the diaphragm in postural mechanisms. Separating the respiratory signal from the postural signal was important in cases when postural movements start to form a large proportion of the diaphragm motion, as in situation 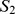 for the pathological group. A simple investigation of the differential curve does not show significant lowering of the respiratory motion range, but after the signals are separated significant changes are revealed in both the postural and the respiratory parts of the movement.

The significant differences in the harmonicity of the diaphragm motion observed in this study indicate changes in the central nervous system related to diaphragm function in subjects with pathological spinal findings suffering from various intensities of chronic low back pain. Low back pain is a wide-spread and widely studied phenomenon. Alternating respiratory patterns and anatomical changes in the diaphragm have been assessed in LBP subjects. Studies concluding increased susceptibility to pain and injury [1], [13], [49] identified differences in muscle recruitment in people suffering from LBP. Janssens [50] used fatigue of inspiratory muscles, and observed altered postural stabilizing strategy in healthy subjects. Jenssens also observed non-worsening stabilization with an already altered stabilizing strategy in subjects suffering from LBP. Grimstone [51] measured respiration-related body imbalance in subjects suffering from LBP, observing worse stability in subjects with LBP. Kolar [44] investigated differences in diaphragm contractions between healthy subjects and LBP subjects. He observed lesser contractions in the posterior part of the diaphragm while the postural demands on the lower limbs increased, and he suspected that intra-abdominal pressure lowering might be the underlying mechanism of LBP. Roussel [34] assessed the altered breathing patterns of LBP subjects during lumbopelvic motor control tests, concluding that some subjects used an altered breathing pattern to provide stronger support for spinal stability.

In our measurements, we did not observe the same diaphragm excursions in the posterior part of the diaphragm for healthy subjects and for subjects suffering from LBP as were observed by [44]. The excursions were reduced in the pathological group. In contrast with Kolar's findings [44], we concluded that there was also lowering of the diaphragm inspiratory position in the pathological group in situation 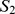. Our measurements support the hypothesis of less diaphragm contraction in the pathological group, with a significant correlation between diaphragm bulging and the intensity of the patient's low back pain. We did not conclude that any other parameters than diaphragm flatness were dependent on the intensity of the subjects' back pain. A high position in the trunk also supports the hypothesis of worse ability to contract the diaphragm in LBP subjects. These findings support the hypothesis that changed diaphragm recruitment would be an important underlying factor for low back pain [33].

In the pathological group, the abdominal muscles lack the ability to hold the ribs in lower position. For this reason, the insertion parts of the diaphragm are not fixed and the diaphragm muscle changes its activation. The diaphragm is disharmonic in its motion, which causes problems with providing respiration and at the same time retaining abdominal pressure. The muscle principle for spine stabilization is therefore violated, and is replaced by a substitute model, which tends more easily toward the emergence of low back pain, spine degeneration or disc hernia.

Reversed causation is always a possibility, i.e. it is possible that the diaphragm behavior is changed in order to stabilize the spine after the deep intrinsic spinal muscles fail. During these changes, breathing patterns may occur, e.g. breath holding and decreased diaphragm excursions. Both of these phenomena were observed in the pathological group in our study. Roussel [34] identified various spinal stability enhancement mechanisms, concluding that a further sub-classification would be needed for the group of LBP patients, according to the variety of spinal supportive mechanisms that they use. Few investigations have been made of diaphragm and breathing patterns, and further research is of basic importance [35].

Some limitations of the the harmonic model of respiratory and postural movements need to be addressed. The modeled breath has to be periodic and preferably harmonious. The breath frequency has to be stable within the observed sequence. If these conditions are not fulfilled the results will be biased. Our measurements were suitable for using the sine model. All subjects displayed stable frequency of breathing. However it is desirable to extend the model to observe the time dependence of the parameters. The sine wave model of diaphragm postural function works well for assessing the range of postural motion. A more complex model needs to be created for a more detailed inspection of the postural function. Magnetic resonance imaging is a reliable method for making detailed observations and assessments of the diaphragm. A restriction of dynamic assessment is the frequency of the movement. This is limited by the sampling (imaging) frequency, which is currently quite low. Thus the diaphragm can be recruited in stabilizing compensation only in static loadings.

### 3.1 Conclusions and future works

Our study shows a way to compare the diaphragm motion within the group of controls without spinal findings and those who have a structural spinal finding, e.g. a hernia, etc., not caused by an injury. In this way, we confirm our experience of the influence of the diaphragm on spinal stability and respiration. The control group show a bigger range of diaphragm motion with lower breathing frequency. The diaphragm also performs better harmonicity (coordination) within its movement. The postural and breathing components are better balanced. This fact is very important for maintaining the intraabdominal pressure, which helps to support the spine from the front. For this reason, it plays a key role in treating back pain, hernias, etc. In the group of controls we also found a lower position of the diaphragm while it was in inspiration position in tidal breathing and also while being loaded. These facts also support the ability of the diaphragm to play a key role in maintaining the good stability of the trunk. It is also important that we are able to separate the phases of diaphragm movement. This supports both the postural function and the breathing function of this muscle due to MR imaging.

Our findings consistently affirmed worse muscle cooperation in the low back pain population subgroup. A clear relation to spinal disorders and low back pain remains inconclusive, but various findings in the literature have been confirmed. Probably the most important conclusion is that there is a need to further address various mechanisms used by patients to compensate deep muscle insufficiency. We have proposed a technique for assessing respiration properties, and have also separated the diaphragm movement that is not linked with respiration.

This study supports our clinical experience, which is based on observations of the difference in motor control of the respiratory and stabilization muscles in patients with and without low back pain. Our clinical experience has indicated that the function is different. This motor control ability to use trunk stabilization muscles needs to be learned by patients with back pain. We believe that diaphragm movements imaging could be a tool for diagnostic support for muscle imbalance in this area. Postural motions of the diaphragm could predict dispositions to vertebrogenic problems or could help when seeking to correct these problems. To verify this suggestion, it is necessary to broaden the group of subjects and to establish a norm for the healthy population.
